# Phenol removal from aqueous solution using *Citrullus colocynthis* waste ash

**DOI:** 10.1016/j.dib.2018.03.049

**Published:** 2018-03-19

**Authors:** Mehdi Qasemi, Mojtaba Afsharnia, Ahmad Zarei, Ali Asghar Najafpoor, Samira Salari, Mahmoud Shams

**Affiliations:** a Department of Environmental Health Engineering, Faculty of Health, Gonabad University of Medical Sciences, Gonabad, Iran Sciences, Mashhad,; b Department of Environmental Health Engineering, School of Health, Mashhad University of Medical Sciences, Mashhad, Iran; c Social Determinants of Health Research Center, Mashhad University of Medical Sciences, Mashhad, Iran

**Keywords:** Phenol, Adsorption, *Citrullus colocynthis*, Thermodynamic, Water

## Abstract

Phenol is a hazardous organic chemical that introduced into the environment by industrial and pharmaceutical discharges. As a versatile option for phenol removal, adsorption would be viable if it accompanying with low cost adsorbents. This article described a natural, very cheap and local available adsorbent for phenol removal. Phenol showed a high affinity to *Citrullus colocynthis* waste ash which mainly composed of SiO_2_ (41.6%), Al_2_O_3_ (17.3%) and MgO (15.9%). Up to 70% of phenol adsorbed in the first 30 min of agitation. The phenol removal was increased by increasing adsorbent dose (0.5–10 g/L) and decreasing pH (2–12) and pollutant concentration (10–100 mg/L). The positive value of **∆***H***°** in thermodynamic data (0.06) revealed that the process is endothermic. The high and positive value of ∆*S*° (13.01) and negative values of ∆*G*° (− 5.36 to − 7.28), showed a high affinity of phenol to the adsorbent and the spontaneous nature of the adsorption. Isotherm modelling revealed that the phenol molecules adsorbed in multilayer with the maximum adsorption capacity of 173.2 mg/g. The rate limiting step in the sorption process was chemisorption, based on the kinetic data.

**Specifications Table**

**Table:** 

Subject area	*Chemical Engineering*
More specific subject area	*Adsorption*
Type of data	*Table, figure*
How data was acquired	*After sorption process, the residual phenol concentrations were determined using DR-5000 spectrophotometry (UV–vis) at 500 nm.*
Data format	*Analyzed*
Experimental factors	*The adsorbent was prepared from a local waste material. Citrullus colocynthis fruit wastes heated at 550 °C for 4 h in the presence of oxygen to produce ash.*
Experimental features	*The adsorption of phenol was investigated as a function of contact time, dose, pH, phenol concentration. Kinetic, isotherm and thermodynamic modeling also presented.*
Data source location	*Gonabad, Khorasan Razavi province, Iran*
Data accessibility	*Data are included in this article.*

**Value of the data**

• Present data described a very cheap and effective waste material for phenol removal from water.
• FTIR and XRD data for *Citrullus colocynthis* waste ash are given.
• Data on the effect of operational variables (contact time, adsorbent dose, pH and phenol concentration) and kinetic, isotherm and thermodynamic models for phenol removal covered.
• The data will be informative to identify the capacity of the adsorbent and rate limiting step of the process.

## 1 Data

Phenol recognized as a priority pollutant by US. Environmental Protection Agency [1]. Wastewater treatment is a key factor to prevent water bodies from being contaminated by phenol and its secondary derivatives [2]. Among the physical, chemical and biological techniques, adsorption considered a very effective, environmental friendly and versatile choice for waste streams treatment [3], [4], [5]. To have a viable prospective in sorption process, many researchers attempt to explore low cost adsorbents for decontamination of polluted waters [6], [7], [8], [9], [10].

On this exploration, we report an adsorbent that prepared from a local available and low cost waste material.

**Fig. 1:**
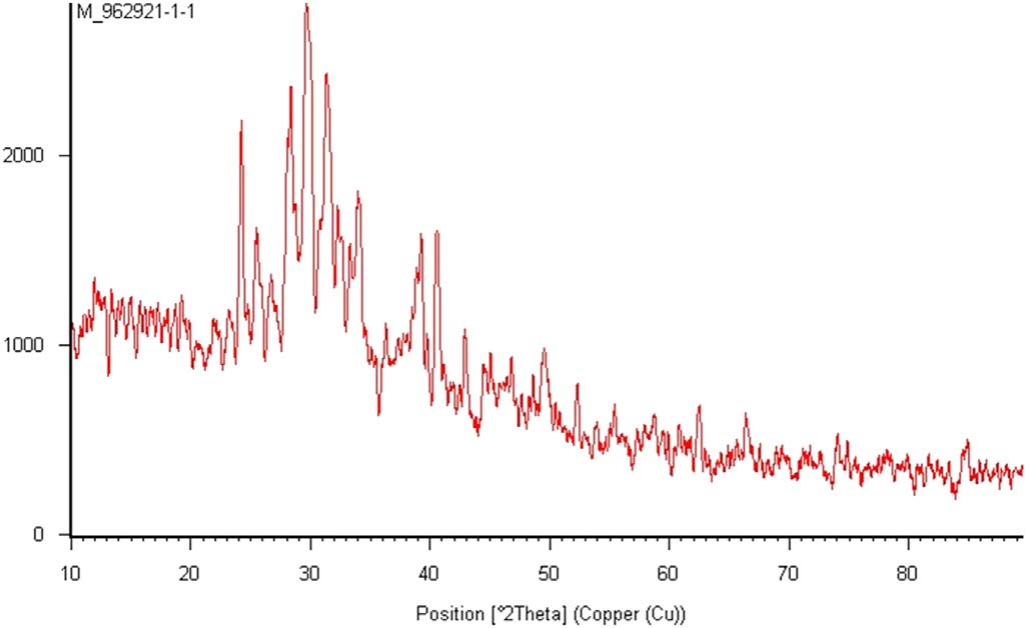
The XRD pattern of the *Citrullus colocynthis* wastes ash.

**Fig. 2:**
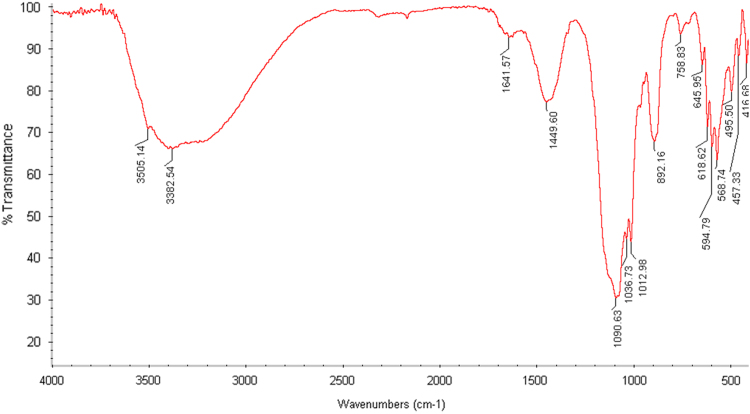
The IR spectra of *Citrullus colocynthis* wastes ash.

The adsorbent was characterized using X-ray fluorescence (XRF) for elemental analysis and X-ray diffraction (XRD) techniques. The elemental composition of *Citrullus colocynthis* wastes ash shown in Table 1. As seen, SiO_2_ and Al_2_O_3_ were among the major chemical constituents of the adsorbent. The XRD pattern of the adsorbent also shown in Fig. 1. The IR spectra of *C. colocynthis* wastes ash in Infrared spectroscopy which is an analytical tool that provides information on the chemical structure of material presented in Fig. 2.

**Table 1:** The XRF analysis of the *Citrullus colocynthis* wastes ash.

**Element**	SiO_2_	Al_2_O_3_	MgO	Fe_2_O_3_	CaO	MnO
**Percent**	41.6	17.3	15.9	11.9	1.6	0.4

## 2 Adsorbent preparation, materials and solutions, experimental design, measurements

To prepare the adsorbent, the *C. colocynthis* fruit wastes washed with deionized water and then dried in oven at 80 °C. The dried materials then heated in furnace at 550 °C for 4 h in the presence of oxygen to produce ash. Next, ash strained using a 20 mesh sieve and kept in a dry environment for following use.

All the experiments were performed in batch sorption mode on aqueous phenol solutions. After adjusting the variable parameters for each run, the solution filtered and then analyzed for residual phenol. The phenol concentration was determined by spectrophotometer in 500 nm according to the standard methods for the examination of water and wastewater [12]. The removal efficiency then calculated using the following equation:

(1) $$\mathrm{Removal}\left(\%\right)=(\frac{C_{0-}C_{f}}{C_{0}})\times 100$$

In which *C_0_* and *C_f_* are the initial and final concentration of phenol.

## 3 The effect of adsorbent dose

**Fig. 3:**
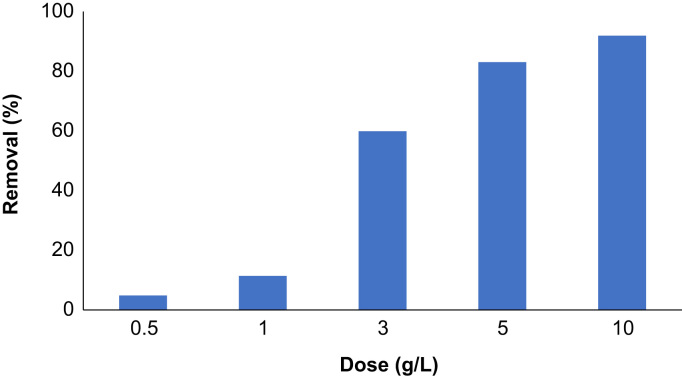
Phenol removal as a function of adsorbent dose (phenol: 50 mg/L, time: 60 min).

Adsorbent dose is an important parameter in the sorption process which determine the available sites for adsorbate attachment to the surface. In this work, the removal efficiency determined by adjusting the adsorbent dose in the range of 0.5–10 g/L. As shown in Fig. 3, the phenol removal increased considerably by adsorbent dose and considerable increase in removal observed when the adsorbent dose adjusted beyond 3 g/L.

## 4 The effect of initial phenol concentration

**Fig. 4:**
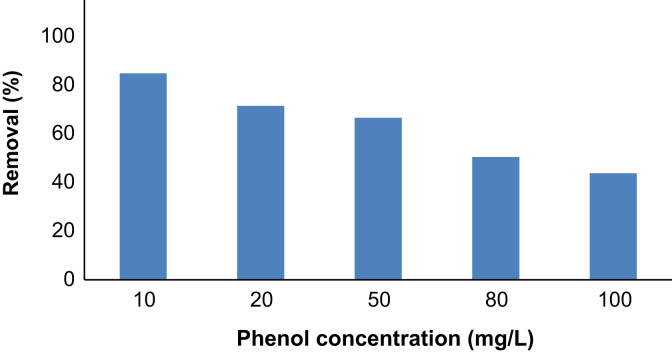
Phenol removal as a function of initial concentration (adsorbent: 3 g/L, time: 60 min).

The phenol removal efficiency as a function of initial phenol concentration is shown in Fig. 4. As shown, the adsorption decreased by phenol concentration.

## 5 The effect of solution pH

**Fig. 5:**
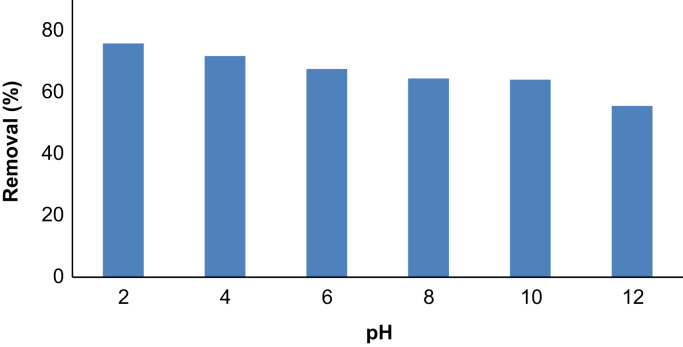
Phenol removal as a function of pH (phenol: 50 ppm, adsorbent: 3 g/L, time: 60 min).

The effect of solution pH in the range of 2–12 on phenol adsorption is shown in Fig. 5. As seen, the removal decreased from about 78% to 58% when pH decreased from 2 to 12.

## 6 Thermodynamic modeling

The effect of solution temperature on adsorption was determined by performing the experiments in temperatures ranged from 20 to 50 °C. Three most important thermodynamic parameters are ∆*H*°, ∆*S*° and ∆*S*° which are standard enthalpy, standard entropy and standard free energy, respectively. The following equations were used to calculate these parameters [13]:

(2) $$∆G{}^{\circ}=-\mathrm{RT}\;\ln\;K_{L}$$

(3) $$\ln\;K_{L}=\frac{\Delta S}{R}\;-\;\frac{∆H{}^{\circ}}{\mathrm{RT}}$$

In the Eqs. (2), (3), *K_L_*, *R* and *T* are the Langmuir constant (L/mg), the universal gas constant (8.314 J/mol K) and the absolute temperature of the solution (*K*). The increasing the adsorption efficiency by temperature as shown in Fig. 5 as well as the positive sign of **∆***H***°** in Table 2, indicates that the process is endothermic in nature.

**Table 2:** Thermodynamic parameters of phenol adsorption.

**Temperature (K)**	**Ce (mg/L)**	**∆*G*° (kJ mol**^**−1**^**)**	**∆*H*°**	**∆S°**
293	18.12	−5.365	0.062714	13.01442
303	15.1	−6.016		
313	13.4	−6.538		
323	11	−7.285		

**Fig. 6:**
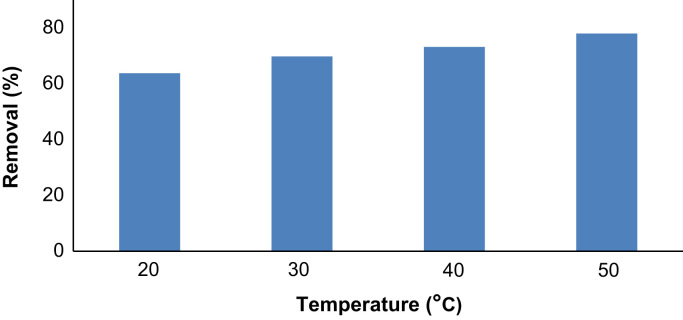
Phenol removal as a function of temperature (phenol: 50 ppm, adsorbent: 3 g/L, time: 60 min).

**Fig. 7:**
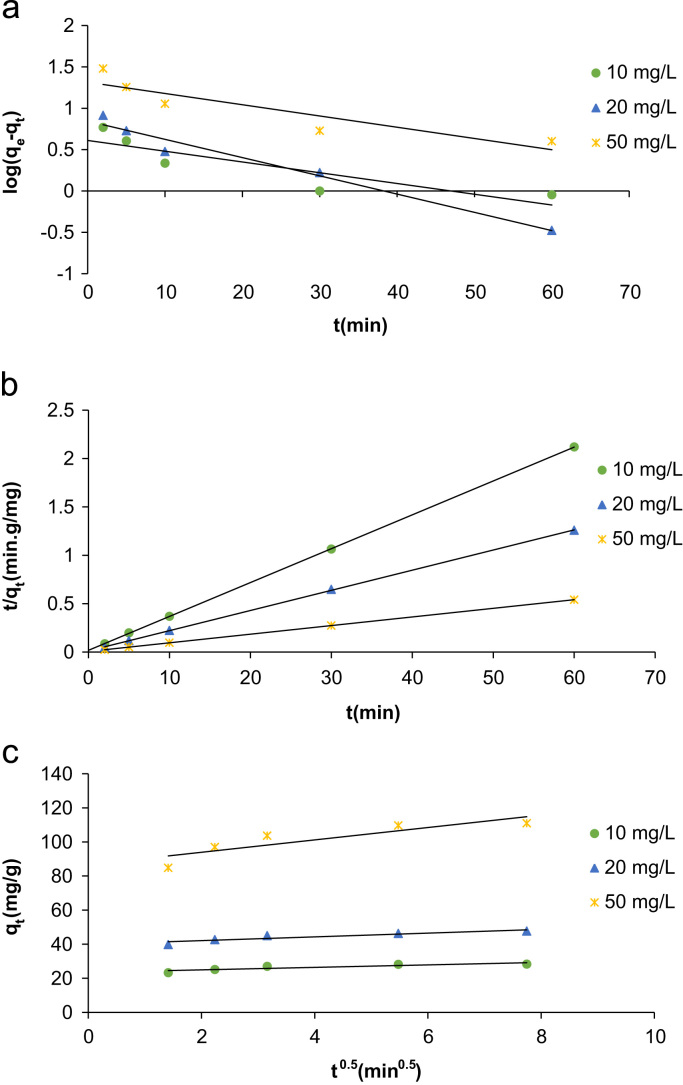
Fitting the experimental data with the (a) Pseudo-first-order, (b) Pseudo-second-order and (c) Intraparticle diffusion kinetic model.

**Fig. 8:**
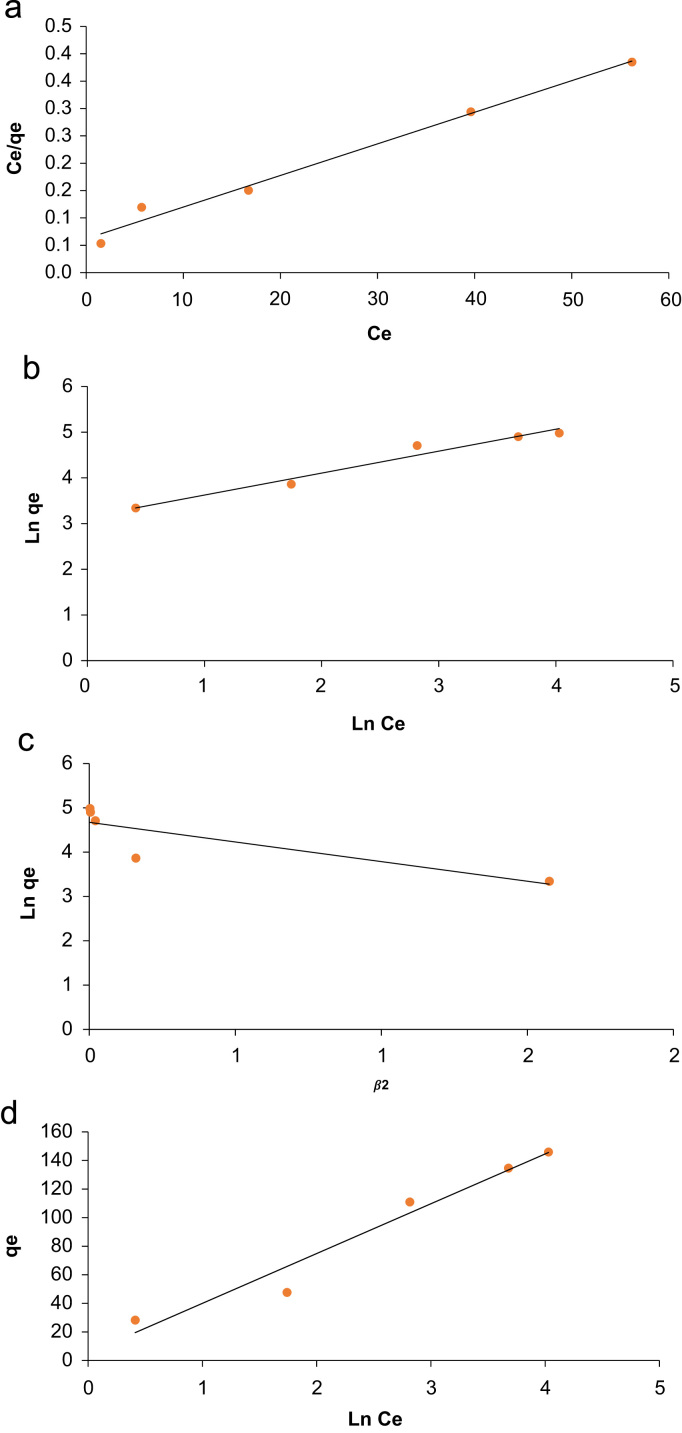
Fitting the experimental data with (a) Langmuir, (b) Freundlich, (c) Dubinin–Radushkevich and (d) Temkin models.

The high and positive value of ∆*S*° in thermodynamic experiments showed a high affinity of phenol to the adsorbent and the increasing randomness during the sorption process. Negative sign of ∆*G*° also revealed that the adsorption is spontaneous (Fig. 6, Fig. 7, Fig. 8).

## 7 Kinetic modeling

Kinetic modeling is an important part of a sorption process that investigate the sorption rate in time. This parameter is important in the economy of the process because it determine the volume of real sorption reactors. The data fitted with three most used models that presented in Table 3. The higher conformity coefficient (*R*^2^) for Pseudo-second-order kinetic model in this experiments (Table 4), indicate that the rate of sorption process controlled by chemisorption.

**Table 3:** Kinetic models used for phenol adsorption [11].

**Kinetic model**	**Formula**	**Plot**
Pseudo-first-order kinetic model	$\mathrm{Log}\left(q_{e}-q_{t}\right)=\log\;q_{e}-\frac{k_{1}}{2.303}.t$	log(*q_e_*–*q_t_*) vs. *t*
Pseudo-second-order kinetic model	$\frac{t}{q_{t}}=\frac{1}{k_{2}\;\;q{е}^{2}}+\frac{1}{qе}.t$	$\frac{t}{q_{t}}$ vs. *t*
Intraparticle diffusion kinetic model	$q\text{t}=k_{p}.t^{0.5}+c$	*q_t_* vs. *t*^0.5^

**Table 4:** Constants obtained from kinetic models for TC adsorption.

*C_0_*[mg/L]	*q_e, exp_* [mg/g]	**Pseudo-first order**			**Pseudo-second order**			**Intra-particle diffusion**	
		*q_e,cal_* [mg/g]	*K*_1_ [min^−1^]	*R*^2^	*q_e,cal_* [mg/g]	*K*_2_ [min^−1^]	*R*^2^	*K_p_* [mg/g min^−0.5^]	*R*^2^
10	85	4.09	−0.03	0.76	28.58	0.06	0.99	0.72	0.77
20	48.1	6.99	−0.05	0.96	48.01	0.03	0.99	1.1	0.84
50	111.6	20.64	0.031	0.81	112.4	0.011	0.99	3.62	0.77

## 8 Equilibrium modeling

In general, the higher the capacity of adsorbent toward a specific contaminant, the lower cost for the regeneration of the sorption media. Isotherm equations model the sorption data when the adsorption reached equilibrium. The isotherm equations used in the modeling of phenol removal are summarized in Table 5. The correlation coefficient of 0.99 for Freundlich model indicates that the adsorption occurred in multilayer. The highest monolayer capacity of adsorbent in this experiments was 173.2 mg/g.

**Table 5:** Isotherm models for phenol adsorption [11].

**Isotherm**	**Linear form**	**Plot**	**Parameter**	
Langmuir	$\frac{\mathrm{Ce}}{\mathrm{qe}}=\frac{1}{\mathrm{qm}}\mathrm{Ce}+\frac{1}{\mathrm{qmb}}$	$\frac{C_{e}}{q_{e}}$ vs. $C_{e}$	*q _max_* (mg/g)	173.2
			*K_L_* (L/mg)	0.092
			*R*^2^	0.986
Freundlich	Log $q_{e}$= log $K_{F}$+ $\frac{1}{n}$ log $C_{e}$	$\log\;q_{e}$ vs. $\log\;C_{e}$	*K_F_*(mg/g(L/mg)^1/n^)	1391.8
			*n*	2.083
			*R*^2^	0.967
Temkin	$q_{e}=B_{1}\ln.k_{t}\;+\;B_{1}\ln\;C_{e}$	$q_{e}$ vs. $\ln\;C_{e}$	*k_t_* (L/mg)	0.999
			*B*_1_	34.85
			*R*^2^	0.957
Dubinin–Radushkevich	${\ln\;q}_{e}={\ln\;q}_{m}\;-\;\beta \epsilon^{2}$	$q_{e}$ vs. $\varepsilon^{2}$	*q*_max_ (mg/g)	107.29
			β	8.86
			*R*^2^	0.71
