# Supplementary material for: Phenol removal from aqueous solution using Citrullus colocynthis waste ash
Source: Data Brief. 2018 Mar 19;18:620–8. doi: 10.1016/j.dib.2018.03.049 (PMC5996314; doi:10.1016/j.dib.2018.03.049)
Supplement: Supplementary file 1 — Transparency document [file mmc1.docx]

Data article

**Phenol removal from aqueous solution using Citrullus colocynthis waste ash**

Mehdi Qasemi^a^, Mojtaba Afsharnia^a^, Ahmad Zarei^a^, Ali Asghar Najafpoor^b, c^, Samira Salari^a^, Mahmoud Shams^b, c *^

^a^ Department of environmental health engineering, faculty of health, Gonabad University of Medical Sciences, Gonabad, Iran Department of Environmental Health Engineering, School of Health, Mashhad University of Medical Sciences, Mashhad, Iran

^b^ Department of Environmental Health Engineering, School of Health, Mashhad University of Medical Sciences, Mashhad, Iran

^c^ Social Determinants of Health research center, Mashhad University of Medical Sciences, Mashhad, Iran

*Corresponding author Contact email: Mahmoud Shams ([Shamsmh@mums.ac.ir](mailto:Shamsmh@mums.ac.ir))

**Conflict of Interest:**

The authors of this article declare that they have no conflict of interests.
